# Complement-dependent cytotoxicity of anti-human osteogenic sarcoma monoclonal antibodies.

**DOI:** 10.1038/bjc.1982.244

**Published:** 1982-10

**Authors:** M. R. Price, M. V. Pimm, R. W. Baldwin

## Abstract

Two mouse monoclonal antibodies against the human osteogenic sarcoma 791T were examined for their capacity to exert complement-dependent cytotoxicity against a panel of human tumour cell lines. Cytotoxicity was most evident against the immunizing tumour 791T although significant reactivity was directed against other osteogenic sarcomas. In admixture, the 2 antibodies displayed synergism in their cytotoxicity although this was only demonstrable with defined ranges of antibody concentration. The cytotoxicity of these antibodies was dependent upon the use of rabbit serum as complement and no tumour-cell lysis was produced using human, guinea-pig or mouse serum complement. The more potent cytotoxic antibody failed to modify the outgrowth of 791T tumour xenografts in immunodeprived mice even though localization of antibody at the tumour site has been demonstrated (Pimm et al., 1982).


*Br. J. Cancer* (1982) **44**, 601

### COMPLEMENT-DEPENDENT CYTOTOXICITY OF ANTI-HUMAN OSTEOGENIC SARCOMA MONOCLONAL ANTIBODIES

M. R. PRICE, M. V. PIMM AND R. W. BALDWIN

*From the Cancer Research Campaign Laboratories, University of Nottingham, University Park, Nottingham NG7 2RD*

Received 2 April 1982 Accepted 24 May 1982

**Summary.—Two mouse monoclonal antibodies against the human osteogenic sarcoma 791T were examined for their capacity to exert complement-dependent cytotoxicity against a panel of human tumour cell lines. Cytotoxicity was most evident against the immunizing tumour 791T although significant reactivity was directed against other osteogenic sarcomas. In admixture, the 2 antibodies displayed synergism in their cytotoxicity although this was only demonstrable with defined ranges of antibody concentration. The cytotoxicity of these antibodies was dependent upon the use of rabbit serum as complement and no tumour-cell lysis was produced using human, guinea-pig or mouse serum complement. The more potent cytotoxic antibody failed to modify the outgrowth of 791T tumour xenografts in immunodeprived mice even though localization of antibody at the tumour site has been demonstrated (Pimm *et al.*, 1982).**

SINCE THE INTRODUCTION of hybridoma technology (Köhler & Milstein, 1975), monoclonal antibodies have been produced which show preferential reactivity with malignant cells, although their potential as effective therapeutic agents has yet to be realized (reviewed by Baldwin *et al.*, 1981). The powerful cytotoxicity of IgM and IgG2 monoclonal antibodies in the presence of complement suggests a direct role for these antibodies in limiting tumour development or controlling residual disease. Already, cytotoxic murine antibodies have been prepared against a number of human tumours, and therapeutic manipulations using the passive administration of antibody have been attempted in immunodeficient mice bearing human tumour xenografts (Herlyn *et al.*, 1980; Herlyn & Koprowski, 1981) and with leukaemia in mice (Bernstein *et al.*, 1980) and humans (Ritz *et al.*, 1981; Miller *et al.*, 1981) with varying degrees of success.

In preliminary tests, it was evident that the 2 anti-human osteogenic sarcoma monoclonal antibodies described by Embleton *et al.* (1981*a*, *b*) exhibited complement-dependent cytotoxicity against the immunizing osteogenic sarcoma cell line, 791T. In the present investigation, the capacity of these antibodies to mediate complement-dependent cytotoxicity against a panel of osteogenic sarcomas and other tumours has been evaluated using a short-term $^{51}Cr$-release test. An important aspect of this work was to determine whether the anti-tumour cytotoxic reactions were demonstrable using mouse and human sera as sources of complement, since these antibodies have therapeutic potential in model studies with human tumour xenografts in immunodeprived mice or in patients.

### MATERIALS AND METHODS

*Cells.*—Human tumour cell lines employed as target cells in complement-dependent cytotoxicity assays included osteogenic sarcomas (791T, 788T, 278T and 20S), prostate carcinoma (EB33), lung carcinoma (A549) and ovarian carcinoma (PA1). Cell lines were grown as monolayers in Eagle's minimum essential medium supplemented with 10% (v/v) fetal calf serum (FCS). Human peripheral blood mononuclear cells were prepared from heparinized blood by density-gradient centrifugation upon Ficoll–Triosil (Lymphoprep—Flow Laboratories, Irvine, Scotland). Cells, suspended at $2\times10^6$/ml in a 1/200 dilution of PHA (Wellcome Reagents Ltd, London) in RPMI 1640 containing 5% FCS $5\times10^{-5}$M 2-mercaptoethanol and gentamycin (50 u/ml), were dispensed in Costar 24 Well Cluster Plates (A. R. Horwell, London) at 1 mg/ml. The preparation of PHA-blasts was harvested after incubation at 37°C for 48 h and washed twice by centrifugation in Eagle's HEPES medium containing 5% FCS.

*Anti-osteogenic sarcoma monoclonal antibodies.*—Hybridomas 791T/36 Clone 3 and 791T/48 Clone 15 (Embleton *et al.*, 1981*a*, *b*) provided the source of antibody in supernatants from *in vitro* cultures.

Antibody was isolated from culture supernatants by passage through a 5 ml packed bed volume of Sepharose-linked Protein A (Pharmacia, Uppsala, Sweden) at 50 ml/h. The non-bound fraction was discarded and the column was extensively washed with phosphate-buffered saline (PBS) pH 7·3, containing 0·02% (w/v) sodium azide. This column was then connected to a column of Sephadex G25 (Pharmacia) and elution of the bound antibody was achieved by application of 3M NaSCN. The eluted protein peak separated from the NaSCN by Sephadex G25 gel filtration was concentrated by positive-pressure membrane ultrafiltration using a PM10 Amicon membrane (Amicon, High Wycombe). Separate Sepharose–Protein A columns were used for the purification of anti-791T/36 and anti-791T/48 antibodies in order to avoid cross-contamination. The recovery of purified anti-791T/36 and anti-791T/48 antibodies was $16{\cdot}8\pm8{\cdot}0$ and $4{\cdot}3\pm2{\cdot}9$ μg/ml respectively of original hybridoma supernatants. Antibody concentrations were determined using the protein assay of Lowry or spectrophotometrically assuming an $E^{1\%}_{280}$ of 14·3 (Hudson & Hay, 1980). Both monoclonal antibodies were determined as belonging to the mouse IgG2b subclass as defined in immunodiffusion tests using mouse immunoglobulin-typing antisera (Miles Laboratories, Stoke Poges, U.K.).

Anti-HLA–A, B, C (shared determinant) Clone W6/32 HL monoclonal antibody was obtained as serum/ascites fluid (Ig content 2 mg/ml) from Sera-Lab. (Crawley Down, Sussex).

*Complement.*—Lyophilized rabbit serum (Buxted Rabbit Co. Ltd, Sussex) was reconstituted with distilled water immediately before use. Guinea-pig (Dunkin–Hartley strain) serum, CBA/Ca mouse serum and human serum from laboratory personnel was stored at −70°C until use.

*Complement-dependent cytotoxicity assay.*—The complement-dependent cytotoxicity of anti-791T monoclonal antibodies was measured using a 2 h $^{51}$Cr-release assay (Price, 1978). Briefly, tumour target cells were labelled with 125 μCi $Na_2{}^{51}CrO_4$ (Radiochemical Centre, Amersham) for 60 min at 37°C in a volume of 0·5–1·0 ml Eagle's HEPES containing 5% FCS. After washing cells 3× by centrifugation, cells were aliquoted (50 μl containing $10^4$ cells) in quadruplicates into round-bottomed Sterilin M24A microtest plates which contained media, diluted heat-inactivated (56°C for 1 h) sera or diluted monoclonal antibody preparations at 100 μl/well. As source of complement, rabbit, mouse, human or guinea-pig serum (100 μl aliquots) was added to each well at concentrations which had been predetermined for each cell line to be non-toxic for the cells alone. All dilutions were performed using Eagle's HEPES medium containing 5% FCS. After incubation at room temperature for 2 h, plates were centrifuged at 280 *g* for 10 min, and 100μl aliquots of the supernatants were collected and counted for radioactivity using an LKB-Wallac gamma counter. The percentage release of $^{51}$Cr was calculated for each test sample and medium control samples. The maximum percentage release of $^{51}$Cr (>90% with all cell lines) was determined by the addition of 1% sodium dodecyl sulphate to labelled target cells, and the percentage release in samples exposed to medium alone did not exceed 15%. The percentage cytotoxicity values were calculated as [(% $^{51}$Cr release in test samples − % $^{51}$Cr release in medium control samples)/(maximum % $^{51}$Cr release − % $^{51}$Cr release in medium control samples)] × 100%.

*Mice and xenografts.*—CBA/Ca mice (Bantin and Kingman, Hull) were thymectomized at 3–4 weeks of age and 3–6 weeks later received 9 Gy whole-body γ-irradiation from a $^{60}$Co source. The lethal effect of irradiation was prevented by i.p. injection of 200 mg/kg cytosine arabinoside (Cytosar, Upjohn, Crawley) 2 days before irradiation (Steel *et al.*, 1978). Xenograft growths were initiated by s.c. injection of $10^6$–$10^7$ tumour cells harvested with trypsin from *in vitro* cultures and washed in serum-free medium. Mice were maintained on sterile bedding with sterilized food and water and held in isolator cabinets (Vickers Pathoflex Isolator, Basingstoke). They were exsanguinated by heart puncture, and the serum was collected and stored at −20°C. Mice were found to be free of macroscopically visible thymic remnants at the time of blood collection.

### RESULTS

### *Complement-dependent cytotoxicity of anti-human osteogenic sarcoma 791T monoclonal antibodies for various target tumour cell lines*

The anti-human osteogenic sarcoma monoclonal antibodies anti-791T/36 and anti-791T/48 belong to the IgG2b subclass with mouse κ light chain. Murine IgG2b antibodies are complement-fixing (Stanworth & Turner, 1978) and the data in Fig. 1 confirm that both antibodies mediate complement-dependent cytotoxicity against 791T target cells employing rabbit serum as the source of complement. At the highest antibody concentration tested (125 μg/ml), anti-791T/36 monoclonal antibody reacted strongly with the 4 osteogenic sarcoma cell lines 791T, 788T, 278T and 20S, and the cross-reactive prostate carcinoma cell line EB33. This antibody displayed no reactivity for the cell lines A549 (lung carcinoma) and PA1 (ovarian carcinoma). At the lower antibody concentrations, down to 1 μg/ml, it was evident that the cell lines 791T and 788T were the most susceptible to antibody-induced, complement-dependent cell lysis.

The other anti-human osteogenic sarcoma 791T monoclonal antibody, anti-791T/48, exhibited much lower reactivity in these tests although significant levels of cytotoxicity were recorded against the line 791T. This finding is in accord with those of preliminary experiments in which

FIG. 1. Complement-dependent cytotoxicity of anti-osteogenic sarcoma 791T monoclonal antibodies against various human tumour cell lines. The % cytotoxicities of anti-791T/36 and anti-791T/48 antibodies in the presence of rabbit serum complement (C′), at the dilution stated in parentheses, are indicated by solid circles and squares, respectively. In the presence of heat-inactivated (56°C for 60 min) complement, their cytotoxicities are indicated by open circles and squares, respectively. The cytotoxicities of complement alone and heat-inactivated complement alone are presented by solid and open triangles, respectively. The s.d. on all cytotoxicity values >5% (with the exception of one determination) did not exceed 10% of the mean value.

anti-791T/48 hybridoma supernatants failed to induce complement-dependent cytotoxicity. In such supernatants, antibody concentrations are approximately 5 μg/ml, which clearly is insufficient for demonstrable cytotoxicity (Fig. 1).

All cytotoxic reactions against the various cell lines in Fig. 1 were dependent upon the presence of active rabbit serum complement, and all cytotoxic values determined in the presence of heat-inactivated complement (56°C for 1 h) were in the range −0·5 to 1·9% cytotoxicity.

TABLE I.—*Synergistic antibody-mediated, complement-dependent cytotoxicity of anti-791T/36 and anti-791T/48 monoclonal antibodies for osteogenic sarcoma 791T target cells*

| % cytotoxicity (mean ± s.d.) | | | | | | | |
|---|---|---|---|---|---|---|---|
| Concentration of anti-791T/48 monoclonal antibody (μg/ml) | Concentration of anti-791T/36 monoclonal antibody (μg/ml) | | | | | | |
| | 0 | 0·04 | 0·2 | 1 | 5 | 25 | 125 |
| 0 | 0·8±0·5*<br>(0·1±0·7)† | 0·0±0·4<br>*0·0*‡ | 0·0±1·6<br>*0·0* | 3·2±0·6<br>*3·2* | 13·1±2·3<br>*13·1* | 42·6±1·7<br>(1·0±0·7)<br>*42·6* | 64·7±2·2<br>(0·8±0·8)<br>*64·7* |
| 0·2 | 1·1±0·5<br>*1·1* | 0·7±0·5<br>*1·1* | 2·0±0·7<br>(0·4±0·6)<br>*1·1* | 4·7±0·5<br>*4·3* | 16·0±1·6<br>*14·2* | 45·5±2·0<br>*43·7* | 75·4±2·6<br>*65·8* |
| 1 | 0·0±0·2<br>*0·0* | 0·8±1·2<br>*0·0* | 1·6±0·6<br>*0·0* | 5·8±0·9<br>(0·1±0·8)<br>*3·2* | 14·6±0·7<br>*13·1* | 44·0±0·6<br>*42·6* | 76·8±4·3<br>*64·7* |
| 5 | 2·1±0·9<br>*2·1* | 0·9±1·2<br>*2·1* | 2·0±0·7<br>*2·1* | 6·6±1·0<br>*5·3* | 17·9±2·5<br>(−0·1±0·4)<br>*15·2* | 41·1±3·2<br>*44·7* | 76·9±4·3<br>*66·8* |
| 25 | 1·5±0·5<br>*1·5* | 2·3±0·7<br>*1·5* | 3·9±1·8<br>*1·5* | 11·4±0·5<br>*4·7* | 29·1±1·7<br>*14·6* | 50·2±2·1<br>(−0·5±0·1)<br>*44·1* | 81·1±6·6<br>*66·2* |
| 125 | 7·6±1·1<br>(0·3±0·8)<br>*7·6* | 21·1±2·9<br>*7·6* | 30·0±1·6<br>*7·6* | 42·6±2·6<br>*10·8* | 61·5±3·1<br>*20·7* | 70·0±4·4<br>*50·2* | 79·5±1·1<br>*72·3* |

* Upper figures represent the % cytotoxicities of the 2 antibodies in admixture, in the presence of rabbit serum complement (final dilution—1/10).

† Figures in parentheses represent the % cytotoxicities of the 2 antibodies in admixture, in the presence of heat-inactivated rabbit serum complement (final dilution—1/10).

‡ Figures in italics represent the sum of the % cytotoxicities of the 2 antibodies when tested individually.

The pattern of reaction of the 2 monoclonal antibodies with these cell lines parallels those demonstrated using the $^{125}$I–Protein A cell-binding assay (Embleton *et al.*, 1981*a*, *b*).

### *Synergistic, complement-dependent cytotoxicity of anti-791T monoclonal antibodies*

Anti-791T/36 and anti-791T/48 monoclonal antibodies in admixture exhibited synergistic killing of 791T cells when low concentrations of anti-791T/36 antibody (0·04–5 μg/ml) were present with higher concentrations of anti-791T/48 (25 and 125 μg/ml) (Table I). Taking one example, when incubation mixtures contained anti-791T/36 and anti-791T/48 antibodies at 1 and 125 μg/ml respectively, the cytotoxicity measured (42·6 ± 2·6%) was about 4 × the sum (10·8% cytotoxicity) of the 2 antibodies tested separately. The cytotoxic reactions of antibodies in admixture were dependent upon the presence of active rabbit serum complement.

In a second experiment, using the cross-reactive human osteogenic sarcoma cell line 788T, synergistic lysis of target cells was less evident, although it was increased 3-fold (from 16·2 to 43·4% cytotoxicity) with anti-791T/36 and anti-791T/48 at 1 and 125 μg/ml respectively (Table II). The findings with 791T and 788T cells indicate that the 2 antibodies can act synergistically in complement-dependent cytotoxicity tests, although only within a fairly narrow range of concentrations.

### *Effect of source of complement on the cytotoxicity of anti-791T monoclonal antibodies*

While both anti-791T monoclonal antibodies mediated complement-dependent cytotoxicity against 791T cells with rabbit serum complement (Fig. 1 and Tables I and II), neither anti-791T/36 nor anti-791T/48 antibody-induced lysis of 791T cells in the presence of CBA/Ca mouse serum or human serum (individual or pooled samples) as complement. In the

TABLE II.—*Synergistic antibody-mediated, complement-dependent cytotoxicity of anti-791T/36 and anti-791T/48 monoclonal antibodies for osteogenic sarcoma 788T cells.*

| % cytotoxicity (mean ± s.d.) / Concentration of anti-791T/48 monoclonal antibody (μg/ml) | % cytotoxicity (mean ± s.d.): Concentration of anti-791T/36 monoclonal antibody (μg/ml) 0 | 0·04 | 0·2 | 1 | 5 | 25 | 125 |
|---|---|---|---|---|---|---|---|
| 0 | −1·1±0·8*<br>(−1·4±0·1†) | 0·0±0·6<br>*0·0‡* | −0·3±1·5<br>*−0·3* | 12·5±3·3<br>*12·5* | 52·5±3·7<br>*52·5* | 60·2±4·0<br>*60·2* | 64·8±1·2<br>(−0·3±0·4)<br>*64·8* |
| 0·2 | 1·1±1·0<br>*1·1* | 0·0±0·6<br>*1·1* | −0·8±2·4<br>(1·0±1·7)<br>*0·8* | 14·1±2·4<br>*13·6* | 54·0±2·6<br>*53·6* | 58·7±3·6<br>*61·3* | 68·6±3·6<br>*65·9* |
| 1 | 0·6±0·4<br>*0·6* | 0·0±0·4<br>*0·6* | 0·1±0·8<br>*0·3* | 13·3±5·4<br>(1·2±1·0)<br>*13·1* | 54·8±7·3<br>*53·1* | 61·9±4·4<br>*60·8* | 70·2±1·9<br>*65·4* |
| 5 | 1·2±0·6<br>*1·2* | 0·0±0·1<br>*1·2* | 0·1±1·9<br>*0·9* | 13·6±3·2<br>*13·7* | 54·0±3·0<br>(0·6±0·4)<br>*53·7* | 60·9±2·6<br>*61·4* | 68·3±2·2<br>*66·0* |
| 25 | 1·9±1·2<br>*1·9* | −0·3±1·5<br>*1·9* | 0·8±1·2<br>*1·6* | 17·3±5·4<br>*14·4* | 60·9±5·5<br>*54·4* | 66·8±3·5<br>(0·8±1·0)<br>*62·1* | 67·3±4·0<br>*66·7* |
| 125 | 3·7±0·3<br>(−0·3±0·4)<br>*3·7* | 1·9±1·2<br>*3·7* | 4·6±1·1<br>*3·4* | 43·4±1·5<br>*16·2* | 74·5±6·4<br>*56·2* | 73·8±1·0<br>*63·9* | 75·6±3·9<br>(0·3±0·4)<br>*68·5* |

Symbols *, †, ‡, as footnotes to Table I.

TABLE III.—*Complement-dependent cytotoxicity of anti-791T monoclonal antibodies: screen of complement sources*

| Serum source of complement* | % cytotoxicity against: 791T cells treated with: Medium | Anti-791T/36† | Anti-791T/48† | W6/32‡ | PHA-blasts treated with: Medium | W6/32‡ |
|---|---|---|---|---|---|---|
| Rabbit | −0·3±0·3<br>(−0·2±0·1)§ | 53·8±4·7<br>(0·7±0·5) | 29·1±2·8<br>(0·4±0·6) | 46·1±5·3<br>(0·9±0·5) | 8·4±0·8<br>(−0·4±0·6) | 62·3±3·5<br>(0·0±0·2) |
| Mouse | 0·3±0·2<br>(0·2±0·3) | 0·0±0·2<br>(−0·1±0·2) | 0·0±0·1<br>(0·3±0·2) | 0·0±0·3<br>(0·1±0·1) | 0·2±0·7<br>(−0·8±0·7) | 0·4±1·2<br>(0·3±0·4) |
| Human pool | 0·0±0·2<br>(−0·1±0·2) | 0·1±0·1<br>(0·5±0·3) | 0·1±0·1<br>(0·3±0·2) | −0·3±0·2<br>(−0·3±0·2) | 2·1±0·6<br>(−0·3±1·0) | 76·3±0·9<br>(0·6±0·9) |
| Human (1) | 0·0±0·4<br>(0·0±0·3) | 0·1±0·2<br>(0·4±0·3) | 0·4±0·5<br>(0·7±0·6) | 0·0±0·4<br>(0·1±0·1) | 0·6±0·7<br>(0·4±0·3) | 67·6±5·5<br>(0·6±0·6) |
| Human (2) | 0·2±0·5<br>(0·3±0·2) | 0·2±0·1<br>(−0·2±0·3) | 0·3±0·2<br>(0·1±0·4) | −0·5±0·2<br>(−0·6±0·3) | 1·0±1·1<br>(−0·3±0·7) | 66·4±7·2<br>(1·2±1·2) |
| Human (3) | −0·3±0·1<br>(−0·1±0·2) | 0·1±0·3<br>(0·6±0·2) | 0·4±0·2<br>(1·0±0·3) | −0·4±0·3<br>(−0·8±0·2) | 1·0±1·0<br>(−0·6±0·9) | 74·8±4·8<br>(0·7±1·3) |
| Guinea-pig | 0·3±0·5<br>(0·0±0·5) | 0·1±0·3<br>(−0·1±0·2) | 0·2±0·2<br>(0·1±0·2) | 0·5±0·2<br>(0·5±0·1) | 1·1±0·4<br>(−0·1±0·3) | 40·7±4·7<br>(0·1±0·7) |

* Final dilution of serum—1/5.
† Anti-791T/36 and anti-791T/48 monoclonal antibodies were tested at a final concentration of 125 μg/ml.
‡ W6/32 monoclonal antibody was tested at a final dilution of 1/500 of the serum/ascites fluid.
§ Figures in parentheses represent % cytotoxicities determined in the presence of heat-inactivated complement.

positive control tests, rabbit serum (but not guinea-pig serum) again served as a potent source of complement and both monoclonal antibodies produced significant cytotoxicity at 125 μg/ml (Table III). To evaluate whether the human, mouse and guinea-pig sera selected exhibited complement activity, tests were performed using the anti-HLA-A, B, C (shared determinant) monoclonal antibody W6/32. As target cells, 791T tumour cells and PHA-induced human peripheral blood lymphocyte blasts were employed (Table III). This antibody was cytototoxic for 791T target cells in the presence of rabbit serum complement, but not with mouse, human or guinea-pig serum. However, lysis of PHA-induced blasts (40·7–76·3% cytotoxicity) was produced by W6/32 using rabbit, human and guinea-pig serum complement (but again, not with mouse serum complement). In all cases, the cytotoxic reactions were shown to be complement-dependent, and all cytotoxicity values determined in the presence of heat-inactivated (56°C for 1 h) complement were between −0·8 and 1·2% (Table III). At the dilution of complement-source serum used (final dilution 1/5 in all tests), there was little or no evidence of natural cytotoxic activity (due to allo- or heterophile antibodies) against 791T cells or PHA-induced blasts, except that rabbit serum produced a low, complement-dependent cytotoxicity of 8·4 ± 0·8% against PHA-induced blasts (Table III).

*Lytic activity of sera from mice bearing 791T tumour xenografts*

791T tumour cells when injected s.c. into adult thymectomized, γ-irradiated and cytosine-arabinoside-treated CBA/Ca mice developed into progressively growing tumours which kill the recipients after about 1 month (Pimm *et al.*, 1982). The sera from these mice were examined for anti-791T cytotoxic activity and it was determined that between 35 and 40% of mice produced potent cytotoxic antibodies against 791T cells (Fig. 2). All serum samples were tested at a final dilution of

FIG. 2.—Complement-dependent cytotoxicity of 791T-xenograft-bearer serum for 791T tumour target cells. Heat-inactivated serum samples from 30 individual tumour-bearing mice were tested at a final dilution of 1/80, and several of the samples (unselected) were tested additionally at final dilutions of 1/40 and 1/160. All sera were examined for cytotoxicity in the presence of heat-inactivated complement, and in this case all cytotoxic values were in the range −0·4 to 0·7%. Complement—normal rabbit serum at a final dilution of 1/10.

1/80 and some samples (unselected) were also assayed at 1/40 and 1/160; each of the cytotoxic reactions was determined to be complement-dependent and the cytotoxicity of non-tumour-bearer, immunodeprived CBA/Ca mouse serum (6 samples from individual mice and 3 pooled serum samples) was less than 0·8% at each of the dilutions examined. However, the cytotoxicity of these 791T-xenograft-bearer sera was dependent upon the use of rabbit and not CBA/Ca mouse serum as complement (Table IV) and, as shown in Table V, the cytotoxicity was directed against the cell lines 791T, 788T and A549. Since the latter is unreactive with anti-791T/36 and anti-791T/48 antibodies (Fig. 1 and Table V), this tumour-bearer

TABLE IV.—*Complement-dependent cytotoxicity of* 791*T tumour bearer serum* (*TBS*) *for* 791*T tumour cells using rabbit and mouse serum as complement*

| Serum | Final serum dilution | Percentage cytotoxicity using: Rabbit serum complement* | | Mouse serum complement* | |
|---|---|---|---|---|---|
| Medium | — | 0·5±0·1 | (0·1±0·5)† | 0·2±0·2 | (0·2±0·4) |
| 791T TBS (Z547/1)‡ | 1/160 | 6·5±1·3 | (−0·1±0·4) | −0·5±0·2 | (−0·2±0·2) |
| 791T TBS (Z547/2) | 1/80 | 45·9±5·5 | (0·0±0·6) | −0·1±0·2 | (0·1±0·3) |
| 791T TBS (Z555/1) | 1/80 | 64·4±1·6 | (0·9±0·7) | 0·1±0·3 | (0·0±0·4) |

* Final dilution of serum complement—1/5.
† Figures in parentheses represent the % cytotoxicity values determined in the presence of heat-inactivated complement.
‡ Numbers prefixed by "Z" represent Departmental serum code numbers.

TABLE V.—*Complement-dependent cytotoxicity of* 791*T tumour-bearer serum for various tumour target cell lines*

| Serum/antibody | Final dilution/ concentration | % cytotoxicity against 791T cells | 788T cells | | A549 cells |
|---|---|---|---|---|---|
| Medium | — | 0·1±0·1 (0·1±0·1)* | −0·3±0·2 | (−0·1±0·3) | 1·6±0·4 (0·0±0·2) |
| Anti-791T/36 | 25 μg/ml | 40·8±5·1 (0·5±0·4) | 44·6±0·8 | (0·4±0·9) | 1·2±0·1 (0·1±0·6) |
| | 5 μg/ml | 34·3±2·2 | 43·8±4·5 | | 0·7±0·6 |
| | 1 μg/ml | 30·9±4·2 | 36·2±4·7 | | 1·4±0·5 |
| NMS (CBA/Ca) | 1/40 | 0·8±0·5 (0·2±0·1) | | | |
| | 1/80 | 0·3±0·3 | 0·5±0·6 | (0·2±0·2) | 1·3±0·2 (0·5±0·1) |
| | 1/160 | 0·1±0·2 | −0·1±0·6 | (−0·4±0·3) | 1·0±0·3 (0·1±0·4) |
| 791T TBS (Z547/1)† | 1/40 | 25·4±5·1 (0·4±0·2) | | | |
| | 1/80 | 21·3±2·2 | 24·5±3·0 | (0·3±0·4) | 66·6±1·2 (1·9±0·6 |
| | 1/160 | 14·3±2·1 | 0·2±0·2 | (0·2±0·2) | 51·3±1·6 (0·5±0·8 |
| 791T TBS (Z547/2) | 1/40 | 64·8±4·7 (0·5±0·3) | | | |
| | 1/80 | 58·3±2·7 | 60·7±0·1 | (0·4±0·6) | 50·9±4·8 (1·3±0·2) |
| | 1/160 | 45·3±4·7 | 50·0±4·1 | (0·4±0·9) | 12·9±0·1 (0·2±0·8) |
| 791T TBS (Z548/5) | 1/40 | 19.1±2·8 (0·3±0·3) | | | |
| | 1/80 | 12.1±3·3 | 2·9±1·4 | (0·3±0·4) | 13·1±2·0 (0·3±0·6) |
| | 1/160 | 5.0±0·3 | 0·8±0·3 | (0·1±0·4) | 3·5±0·6 (0·2±0·4) |

* Figures in parentheses represent the % cytotoxicity values determined in the presence of heat-inactivated complement (normal rabbit serum, final dilution—1/10).
† Numbers prefixed by "Z" represent Departmental serum code numbers.

cytotoxic response is directed against determinants other than, or in addition to, those defined by the 2 anti-791T monoclonal antibodies.

### In vivo *reactivity of anti*-791*T*/36 *monoclonal antibody*

791T-xenografted, CBA/Ca mice were treated with the more potent monoclonal antibody anti-791T/36, using total inocula of 120 and 240 μg/mouse (Figs 3(a) and (b), respectively) administered in 3 equal intraperitoneal doses on Days 0, 7 and 14. However, the outgrowth of tumours in treated mice was essentially equivalent to that in untreated control animals, and there was no statistically significant difference in the incidence of tumours in test mice (8/11) and controls (10/11) (Fig. 3).

### DISCUSSION

The pattern of complement-dependent cytotoxic reactivity of anti-791T/36 and anti-791T/48 monoclonal antibodies for various tumour target cell lines, and using rabbit serum complement, paralleled that defined using an $^{125}$I–Protein A cell-binding assay (Embleton *et al.*, 1981*a*, *b*) and both antibodies showed maximal

FIG. 3.—Treatment of mice bearing s.c. 791T xenografts by i.p. injection anti-791T/36 monoclonal antibody. Antibody was injected into mice on days and in doses as indicated by the arrows. Untreated mice served as controls.

reactivity against the immunizing cell line, 791T (Fig. 1). In addition, in admixture the 2 antibodies mediated synergistic killing of 791T and 788T osteogenic sarcomas, although this was only evident within a restricted range of concentrations (Tables I and II). The synergistic effects observed were not as pronounced as those demonstrated by Hellström *et al.* (1981) using 2 anti-melanoma monoclonal antibodies which together gave up to 70–90% cytotoxicity at antibody dilutions which with either antibody alone had little or no effect. The explanation for this is that in the melanoma studies the 2 antibodies reacted with 2 distinct antigenic determinants upon the same cell-surface molecule, p 97 (Hellström *et al.*, 1981). The 2 anti-osteogenic sarcoma antibodies are, however, considered to be reactive with antigenic determinants on separate cell surface molecules. The patterns of cross-reactions of the 2 antibodies with various tumour target cells are distinct and clearly distinguishable, suggesting that they are reactive with separate surface antigens (Embleton *et al.*, 1981*a*, *b*). It is not clear why synergistic killing occurred only with mixtures of low concentrations of anti-791T/36 and high concentrations of anti-791T/48 antibodies. It is possible that the antigen defined by anti-791T/48 displays restricted lateral mobility within the plane of the surface membrane so that it represents a poor target for cytotoxic reactions which require close proximity of 2 cell-bound IgG antibodies to initiate the complement cascade. The addition of small quantities of the more cytotoxic antibody anti-791T/36, defining a more mobile surface antigen, would then be expected to enhance cytotoxicity in a synergistic manner.

The 2 anti-osteogenic sarcoma monoclonal antibodies were not cytotoxic for 791T cells using mouse, human or guinea-pig serum as complement (Table III). Mouse complement is known to be both weak and labile so that the failure to demonstrate cytotoxicity using this source of complement is not unexpected. Other studies have demonstrated that normal or nude mouse serum complement is ineffective in mediating lysis compared with rabbit, human or guinea-pig serum (Drake *et al.*, 1973; Herlyn & Koprowski, 1981), although murine IgG2b monoclonal antibodies have been shown to fix mouse complement in haemolysis assays (Neuberger & Rajewsky, 1981). In contrast to the anti-human colon carcinoma monoclonal antibodies described by Herlyn & Koprowski (1981) which utilize human complement to effect lysis, the anti-791T antibodies were non-cytotoxic in the presence of human serum (Table III), although one obvious difference is that the anti-colon carcinoma antibodies belong to the IgM immunoglobulin class whereas the anti-791T antibodies are IgG2b. Clearly, the human sera tested as complement sources were not deficient in this activity, since PHA-induced blasts were lysed by the monoclonal antibody W6/32 and these human sera complement sources. However, even this antibody failed to lyse 791T target cells in the presence of human serum complement and again rabbit serum complement was required (Table III).

Before attempting therapy of 791T-xenografted mice using these monoclonal antibodies, the sera from immunodeprived tumour-bearing mice were examined for anti-tumour activity and it was determined that about 30–40% of animals had circulating antibodies which were highly cytotoxic for 791T target cells (Fig. 2). This cytotoxic response was not a "natural" humoral reaction against tumour cells, since non-tumour-bearing immunodeprived mice did not display this activity even though T-deprivation by thymectomy and irradiation has been reported to increase natural anti-tumour activity in mice (Ménard *et al.*, 1977; Colnaghi *et al.*, 1977). The reactivity of the circulating antibodies was directed against surface antigens other than, or in addition to those defined by the anti-791T monoclonal antibodies since these antibodies were cytotoxic for the anti-791T/36 and anti-791T/48-negative cell line, A549 (Table V). They were not cytotoxic using mouse complement (Table IV), so that they would not be expected to limit tumour growth *in vivo* by their cytotoxic reactivity. The presence of these antibodies in a proportion of tumour-bearing mice also prevented the type of therapeutic manipulation attempted by Herlyn & Koprowski (1981), who administered anti-colon carcinoma monoclonal antibody and rabbit serum as complement to nude mice bearing tumour xenografts.

The monoclonal antibody anti-791T/36 was selected for the *in vivo* tests for 3 reasons: at a practical level, it was available in much greater quantities than anti-791T/48, it is the more cytotoxic of the 2 antibodies (Fig. 1) and, finally, tests performed using radiolabelled anti-791T/36 have shown that this antibody localizes *in vivo* in 791T xenografts in immunodeprived mice (Pimm *et al.*, 1982). The latter is an essential property if any direct anti-tumour activity is to be demonstrated although, as illustrated in Fig. 3, the administration of anti-791T/36 antibody to 791T-bearing mice modified neither tumour development nor incidence. This finding would also infer that antibody-dependent cell-mediated cytotoxicity is inoperative with this antibody, tumour and treatment protocol. Such a mechanism has been invoked to account for the inhibition of growth of human colon carcinomas in nude mice by administration of an anti-colon carcinoma IgG2a monoclonal antibody (Bernstein *et al.*, 1980).

The results of this study demonstrate that anti-human osteogenic sarcoma 791T monoclonal antibodies are cytotoxic for tumour cells *in vitro*, although the full expression of their cytotoxicity depends very much upon the use of rabbit serum as the source of complement. With regard to continuing clinical studies which have demonstrated tumour localization of radio-labelled anti-791T/36 antibody preparations by $\gamma$-scintigraphy, the present findings predict that with tumours showing antibody localization no therapeutic benefit will be produced by complement-dependent cytotoxicity reactions. However, antibodies localizing to tumours may well represent suitable vehicles for the delivery of cytotoxic drugs or bacterial or plant toxins to the site(s) of the tumour.

These studies were supported by the Cancer Research Campaign and by a Government Equipment Grant through the Royal Society.

Thanks are expressed to Dr M. J. Embleton for making available the human tumour cell lines and hybridoma supernatants employed in this investigation, and to Dr R. A. Robins for preparing PHA-blasts. Skilful technical assistance was provided by Mrs J. Bullock, Mrs E. Jacobs, Mrs S. J. Gribben and Mr D. G. Fox.

### REFERENCES

Baldwin, R. W., Embleton, M. J. & Price, M. R. (1981) Monoclonal antibodies specifying tumour-associated antigens and their potential for therapy. *Molec. Aspects Med.*, **4**, 329.

Bernstein, I. D., Tam, M. R. & Nowinski, R. C. (1980) Mouse leukemia: therapy with monoclonal antibodies against a thymus differentiation antigen. *Science*, **207**, 68.

Colnaghi, M. I., Ménard, S. & Della Porta, G.

(1977) Natural anti-tumour serum reactivity in BALB/c mice. II. Control by regulator T-cells. *Int. J. Cancer*, **19**, 275.

DRAKE, W. P., UNGARO, P. C. & MARDINEY, M. R. (1973) Passive administration of antiserum and complement in producing anti-EL4 cytotoxic activity in the serum of C57BL/6 mice. *J. Natl Cancer Inst.*, **50**, 909.

EMBLETON, M. J., GUNN, B., BYERS, V. S. & BALDWIN, R. W. (1981*a*) Antitumour reactions of monoclonal antibody against a human osteogenic-sarcoma cell line. *Br. J. Cancer*, **43**, 582.

EMBLETON, M. J., GUNN, B., BYERS, V. & BALDWIN, R. W. (1981*b*) Antigens on naturally occurring animal and human tumors detected by monoclonal antibodies. *Transplant. Proc.*, **13**, 1966.

HELLSTRÖM, I., BROWN, J. P. & HELLSTRÖM, K. E. (1981) Monoclonal antibodies to two determinants of melanoma-antigen p 97 act synergistically in complement-dependent cytotoxicity. *J. Immunol.*, **127**, 157.

HERLYN, D. M. & KOPROWSKI, H. (1981) Monoclonal anticolon carcinoma antibodies in complement dependent cytotoxicity. *Int. J. Cancer*, **27**, 769.

HERLYN, D., STEPLEWSKI, Z., HERLYN, M. & KOPROWSKI, H. (1980) Inhibition of growth of colorectal carcinoma in nude mice by monoclonal antibody. *Cancer Res.*, **40**, 717.

HUDSON, L. & HAY, F. C. (1980) *Practical Immunology*, 2nd Edn. Oxford: Blackwell Sci. Publ. p. 347.

KÖHLER, G. & MILSTEIN, C. (1975) Continuous cultures of fused cells secreting antibody of predefined specificity. *Nature*, **256**, 495.

MÉNARD, S., COLNAGHI, M. I. & DELLA PORTA, G. (1977) Natural anti-tumour serum reactivity in BALB/c mice. I. Characterization and interference with tumour growth. *Int. J. Cancer*, **19**, 267.

MILLER, R. A., MALONEY, D. G., MCKILLOP, J. & LEVY, R. (1981) *In vivo* effects of murine hybridoma monoclonal antibody in a patient with T-cell leukemia. *Blood*, **58**, 1.

NEUBERGER, M. S. & RAJEWSKY, K. (1981). Activation of mouse complement by monoclonal mouse antibodies. *Eur. J. Immunol.*, **11**, 1012.

PIMM, M. V., EMBLETON, M. J., PERKINS, A. C. & 4 others. (1982) *In vivo* localization of anti-osteogenic sarcoma 791T monoclonal antibody in osteogenic sarcoma xenografts. *Int. J. Cancer*, **30**, 75.

PRICE, M. R. (1978) A microassay for the detection of tumour-specific complement-dependent serum cytotoxicity against a chemically induced rat hepatoma. *Transplantation*, **25**, 224.

RITZ, J., PESANDO, J. M., SALLAN, S. E. & 4 others (1981) Serotherapy of acute lymphoblastic leukemia with monoclonal antibody. *Blood*, **58**, 141.

STANWORTH, D. R. & TURNER, M. W. (1978). Immunochemical analysis of immunoglobulins and their sub-units. In *Handbook of Experimental Immunology* (Ed. Weir, 3rd Edn., Vol. 1. Oxford: Blackwell Sci. Publ. p. 6.1.

STEEL, G. G., COURTNEY, V. D. & ROSTOM, A. Y. (1978) Improved immune suppression techniques for the xenografting of human tumours. *Br. J. Cancer*, **37**, 224.


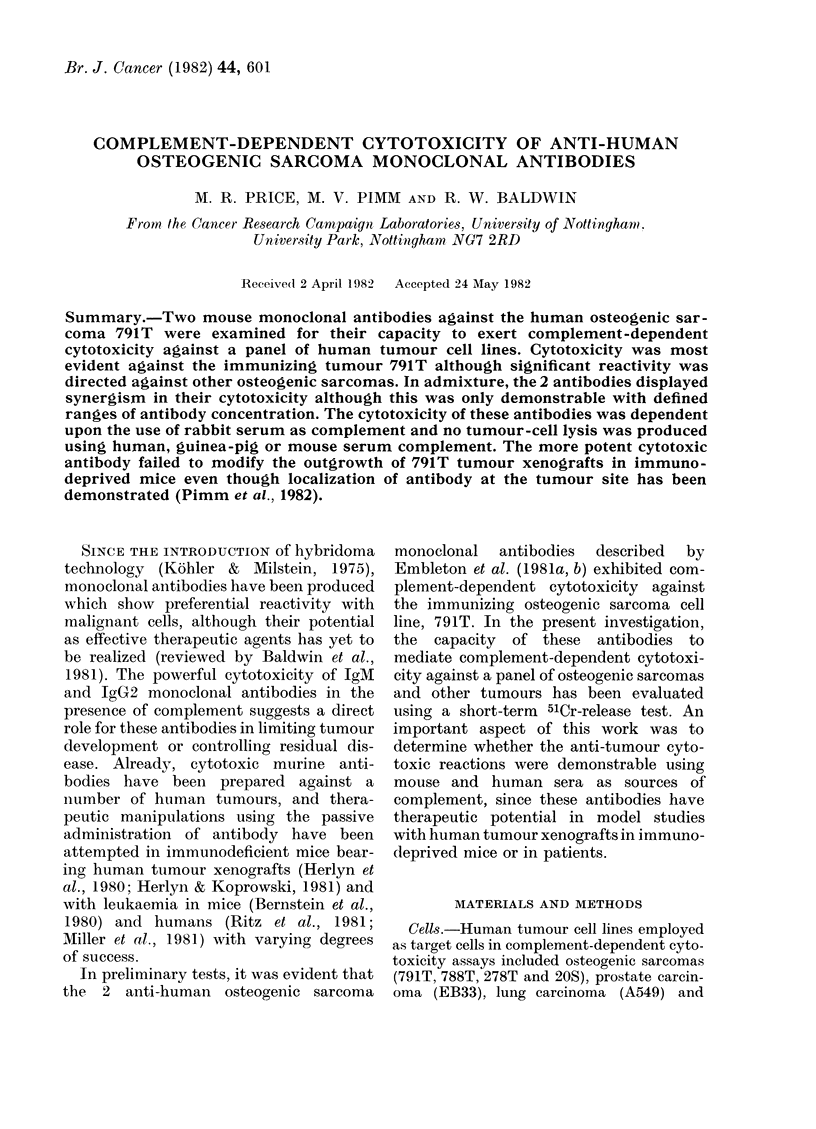

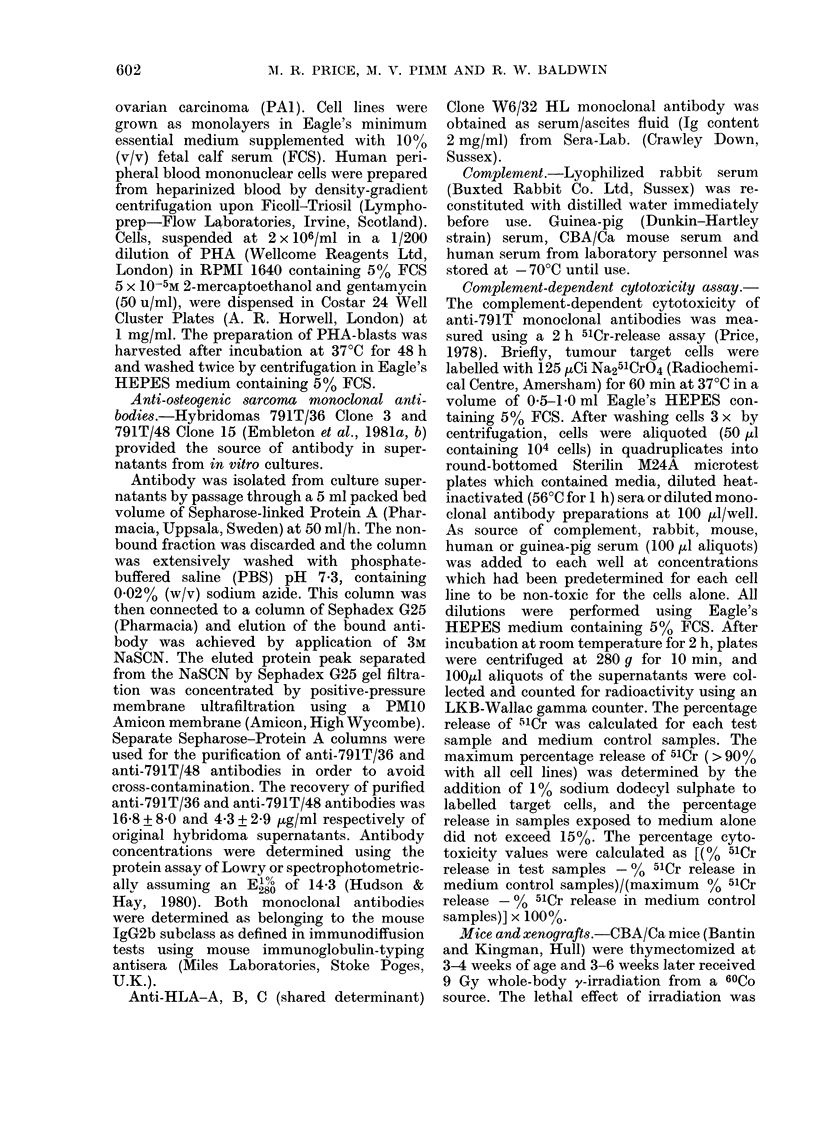

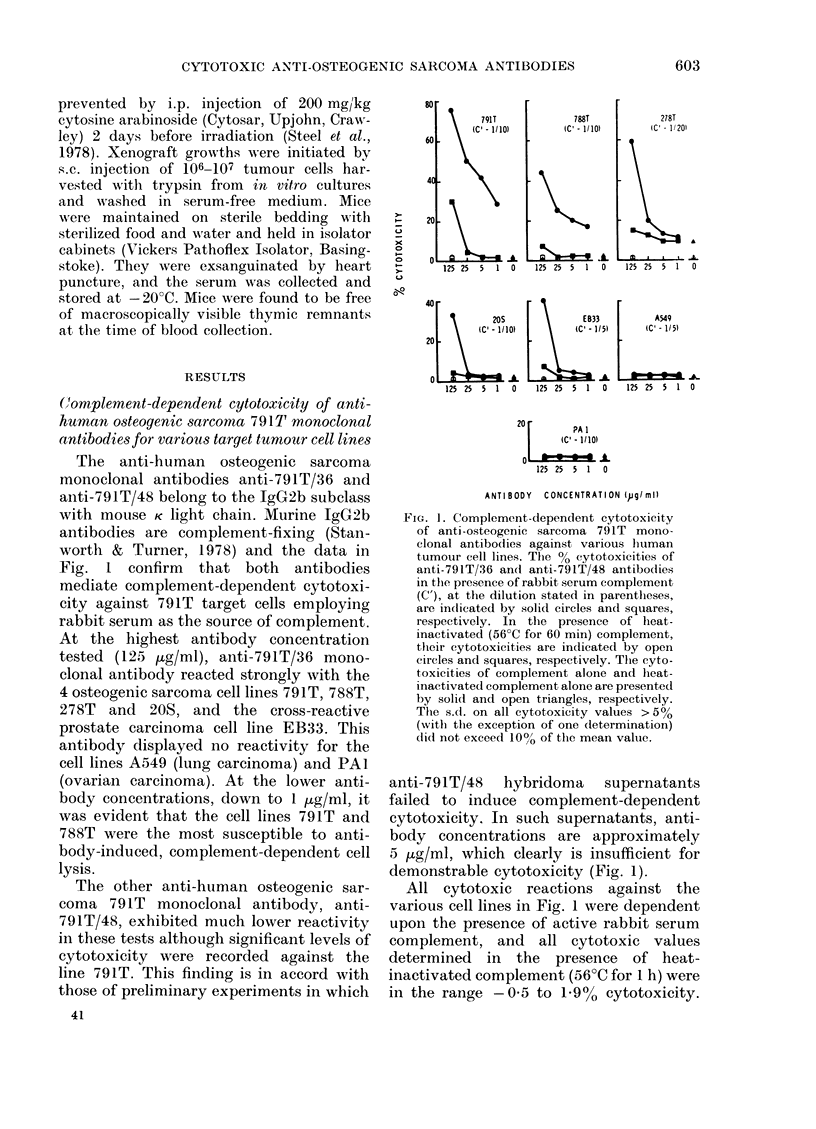

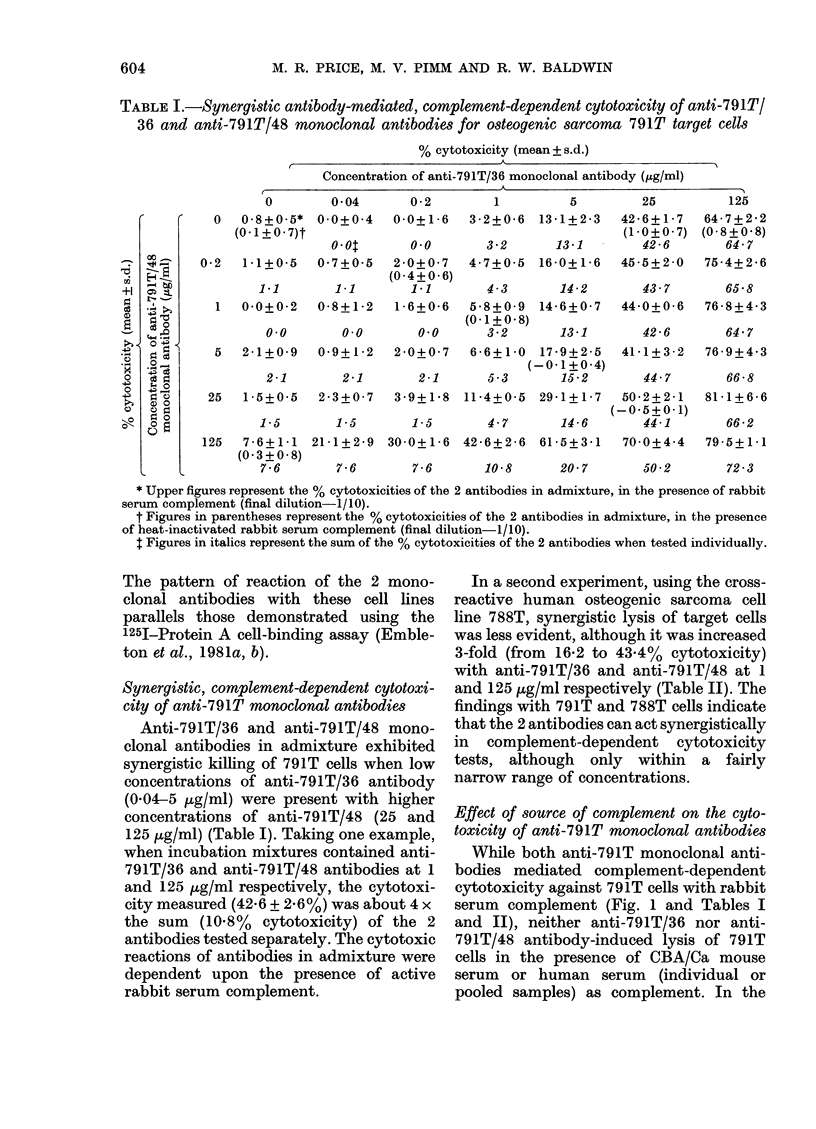

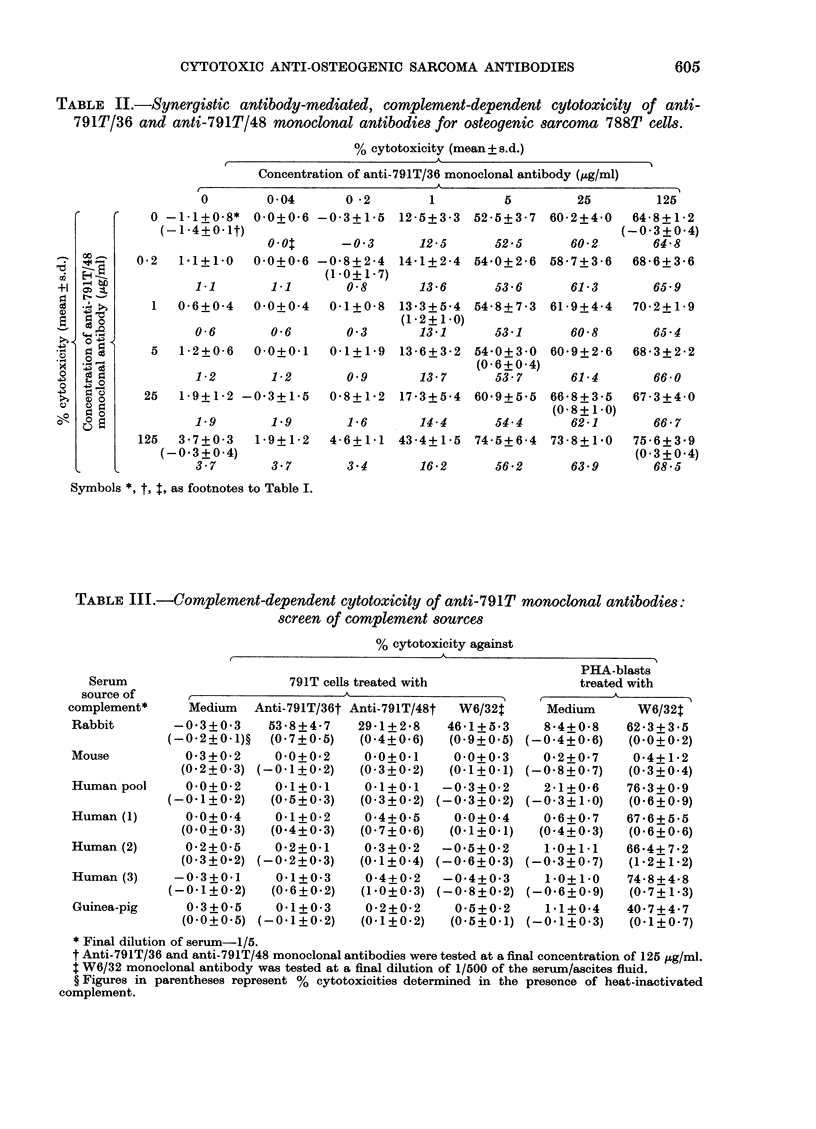

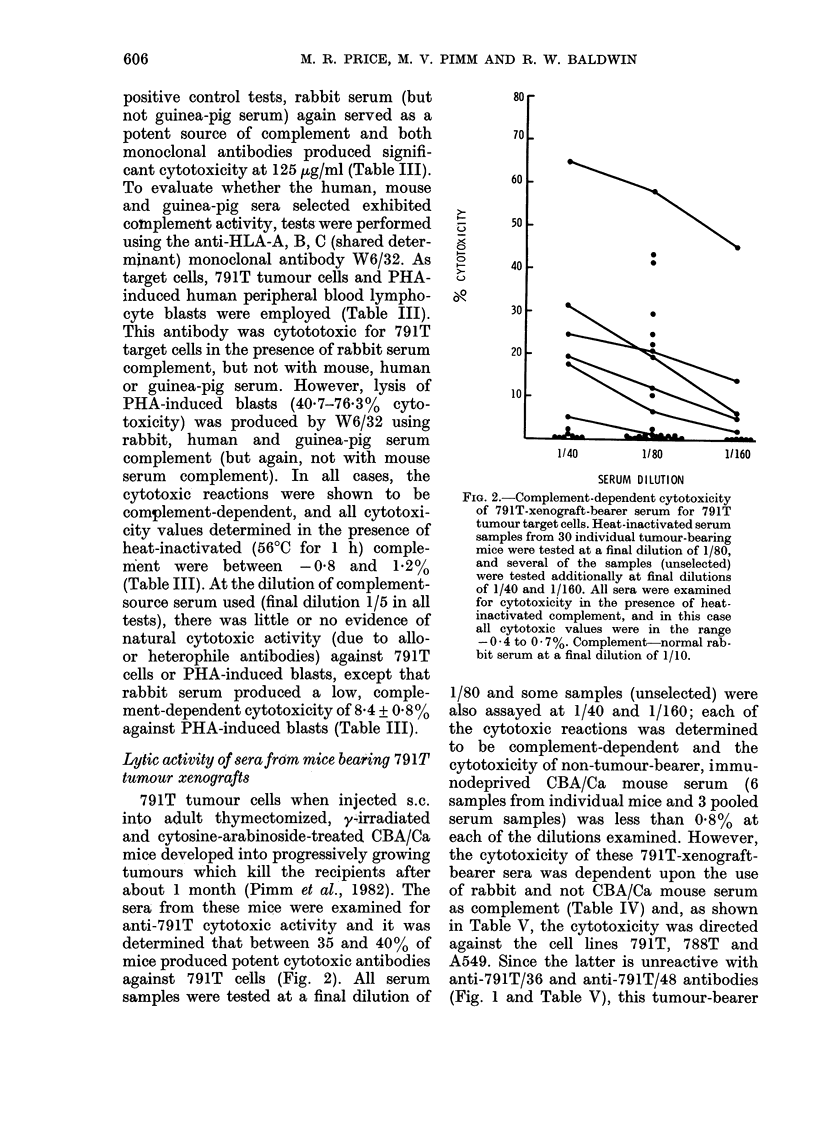

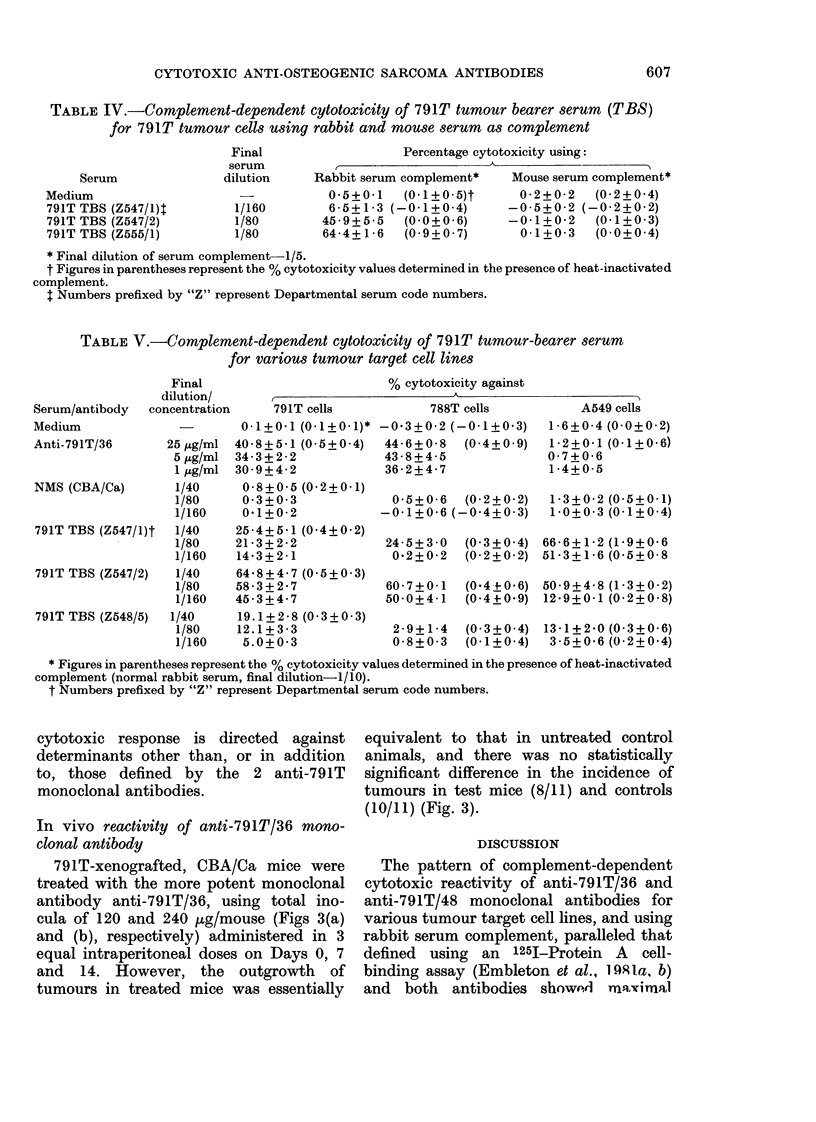

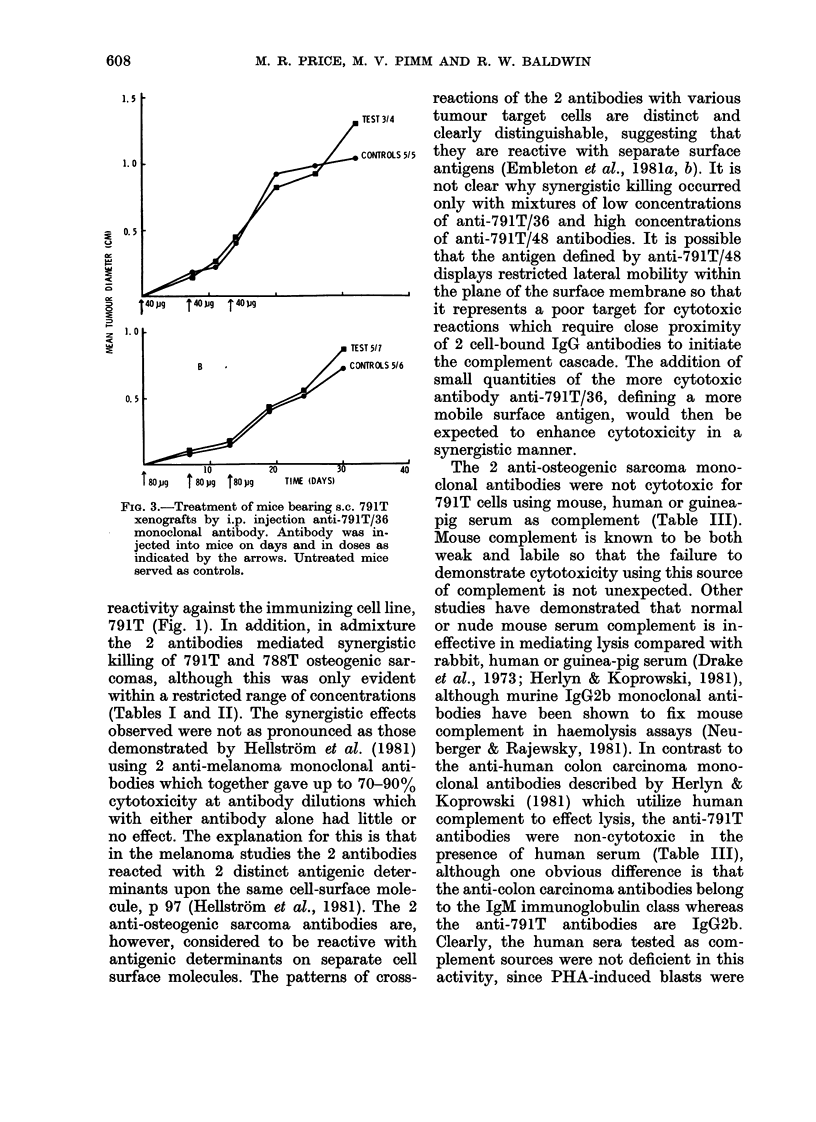

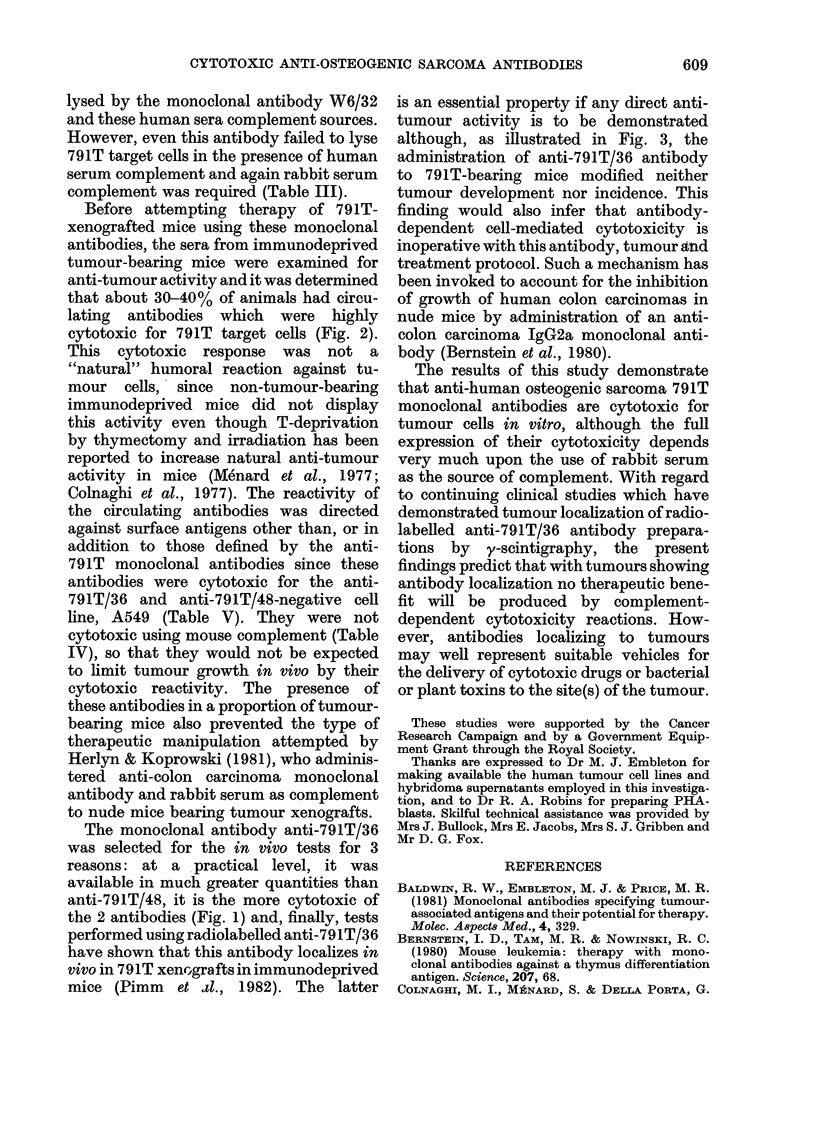

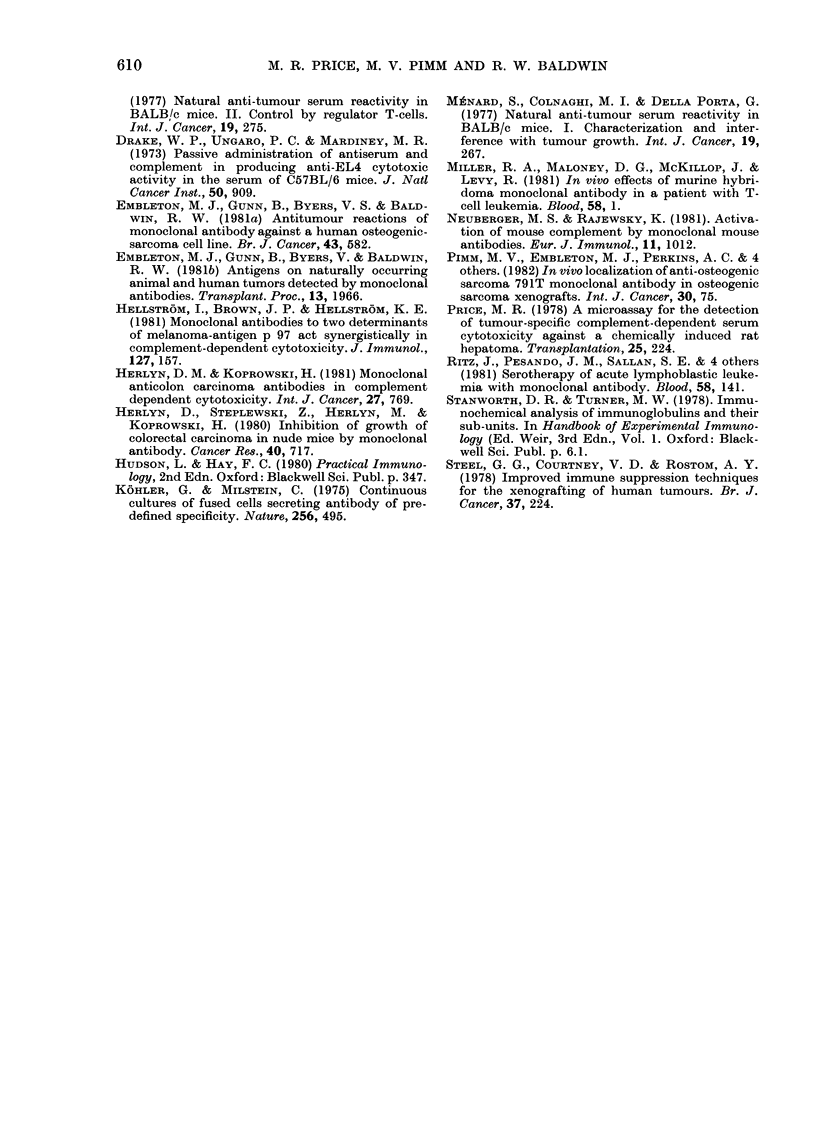


## References

[OCR_01317] Bernstein I. D., Tam M. R., Nowinski R. C. (1980). Mouse leukemia: therapy with monoclonal antibodies against a thymus differentiation antigen.. Science.

[OCR_01331] Drake W. P., Ungaro P. C., Mardiney M. R. (1973). Passive administration of antiserum and complement in producing anti-EL4 cytotoxic activity in the serum of C57BL-6 mice.. J Natl Cancer Inst.

[OCR_01344] Embleton M. J., Gunn B., Byers V. S., Baldwin R. W. (1981). Antigens on naturally occurring animal and human tumors detected by monoclonal antibodies.. Transplant Proc.

[OCR_01340] Embleton M. J., Gunn B., Byers V. S., Baldwin R. W. (1981). Antitumour reactions of monoclonal antibody against a human osteogenic-sarcoma cell line.. Br J Cancer.

[OCR_01350] Hellström I., Brown J. P., Hellström K. E. (1981). Monoclonal antibodies to two determinants of melanoma-antigen p97 act synergistically in complement-dependent cytotoxicity.. J Immunol.

[OCR_01357] Herlyn D. M., Koprowski H. (1981). Monoclonal anticolon carcinoma antibodies in complement-dependent cytotoxicity.. Int J Cancer.

[OCR_01362] Herlyn D. M., Steplewski Z., Herlyn M. F., Koprowski H. (1980). Inhibition of growth of colorectal carcinoma in nude mice by monoclonal antibody.. Cancer Res.

[OCR_01383] Hoyer L. W. (1981). The factor VIII complex: structure and function.. Blood.

[OCR_01371] Köhler G., Milstein C. (1975). Continuous cultures of fused cells secreting antibody of predefined specificity.. Nature.

[OCR_01376] Ménard S., Colnaghi M. I., Porta G. D. (1977). Natural anti-tumor serum reactivity in BALB/c mice. I. Characterization and interference with tumor growth.. Int J Cancer.

[OCR_01389] Neuberger M. S., Rajewsky K. (1981). Activation of mouse complement by monoclonal mouse antibodies.. Eur J Immunol.

[OCR_01400] Price M. R. (1978). A microassay for the detection of tumour-specific complement-dependent serum cytotoxicity against a chemically induced rat hepatoma.. Transplantation.

[OCR_01406] Ritz J., Pesando J. M., Sallan S. E., Clavell L. A., Notis-McConarty J., Rosenthal P., Schlossman S. F. (1981). Serotherapy of acute lymphoblastic leukemia with monoclonal antibody.. Blood.

[OCR_01418] Steel G. G., Courtenay V. D., Rostom A. Y. (1978). Improved immune-suppression techniques for the exongrafting of human tumours.. Br J Cancer.

